# P-193. Risk Factors Associated with Patients Diagnosed with *Clostridioides difficile* Infection with no Documented Antibiotic Exposures

**DOI:** 10.1093/ofid/ofae631.397

**Published:** 2025-01-29

**Authors:** Ana Pranjic, Ursula Patel, Adam K Cheknis, Susan M Pacheco, Larry K Kociolek, Curtis Donskey, Jennifer Cadnum, Stuart Johnson, Matthew H Samore, Dale N Gerding, Charlesnika T Evans, Andrew M Skinner

**Affiliations:** Edward Hines Jr. VA Hospital, Hines, Illinois; Hines VA, Chicago, Illinois; Edward Hines Jr. VA Hospital, Hines, Illinois; Edward Hines, Jr. VA Hospital and Loyola University Medical Center, Hines, Illinois; Ann & Robert H. Lurie Children's Hospital of Chicago, Chicago, IL; Cleveland VA Hospital, Cleveland, Ohio; Northeast Ohio VA Medical Center, Cleveland, Ohio; Hines VA Hospital and Loyola University Medical Center, Hines, Illinois; University of Utah, Salt Lake City, Utah; Edward Hines, Jr. Veterans Affairs Hospital, Hines, Illinois; Northwestern University and VA, Hines, Illinois; University of Utah, Salt Lake City, Utah

## Abstract

**Background:**

*Clostridioides difficile* infection (CDI) is a common healthcare-associated infection leading to significant morbidity and mortality. Antibiotic exposure is a primary risk factor associated with CDI, but other common risk factors for include advanced age, frequent hospitalization, immunosuppression, previous CDI, and gastric acid suppression. This study aimed to evaluate factors associated with CDI with no documented antibiotic exposure.

**Figure:**
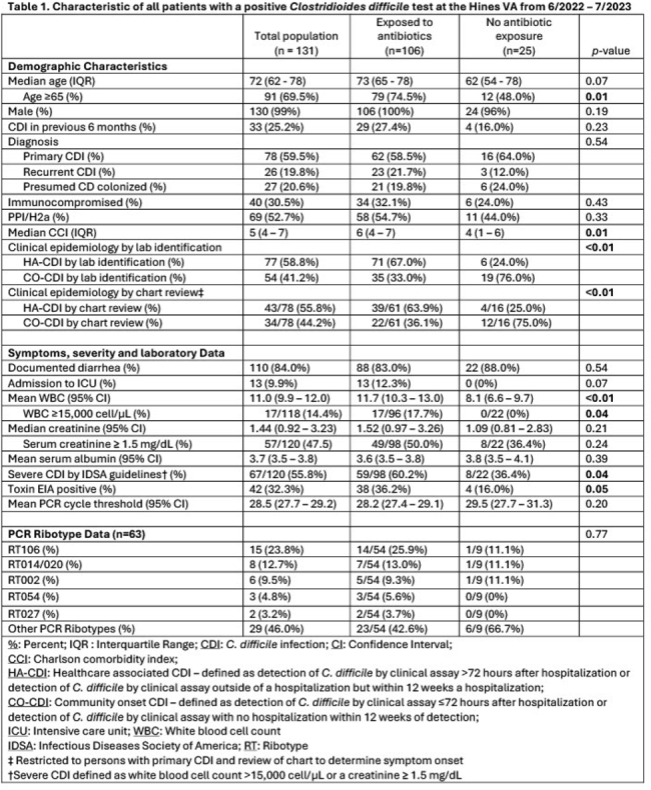

**Methods:**

This is a case-control study of 131 patients with a positive *C. difficile* (CD) test between 7/2022 - 6/2023 at the Edward Hines Jr., VA Hospital. Incident cases were defined as a patient with a positive CD test with no documented antibiotic exposures within 120 days prior to diagnosis, and controls were patients with a positive CD test with documented antibiotic exposures. A multivariable logistic regression model was constructed to determine the adjusted odds ratio (aOR) and 95% confidence intervals (CI) for key CDI risk factors associated with primary CDI and no antibiotic exposure. Available stools were cultured, and recovered CD isolates underwent PCR-ribotyping (RT).

**Figure:**
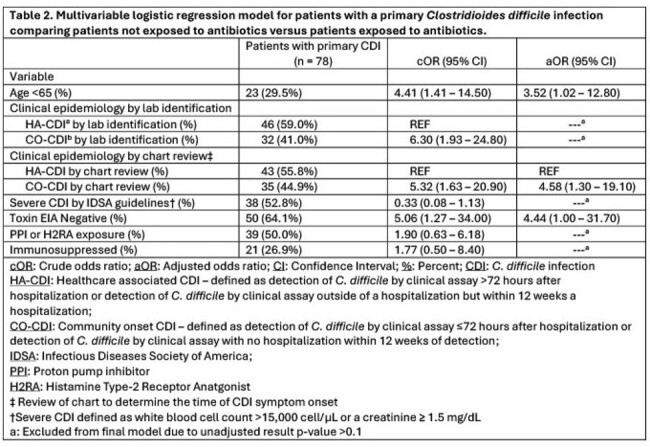

**Results:**

Among all patients, 60% (78/131) had primary CDI, 20% (26/131) had recurrent CDI, and 20% (27/131) were presumed colonized. Among patients with primary CDI, 20% (16/78) had no documented antibiotic exposure. (Table 1) Unadjusted analysis revealed that patients with primary CDI and no antibiotic exposure had increased odds of 1) being age < 65 (crude OR [cOR] 4.41; CI 1.41 – 14.50) 2) having a stool toxin EIA negative test (cOR 5.06; CI 1.27 – 34.00), 3) being classified as a community onset CDI (CO-CDI) (cOR 5.32; CI 1.63 – 20.90). After adjusting for potential confounding variables, patients had increased odds of being age < 65 (aOR 3.52; CI 1.02 – 12.80) and being classified as a CO-CDI (aOR 4.58; CI 1.30 – 19.10). (Table 2) RT106 was the most common strain identified, but there was no strain type associated with a lack of antibiotic exposure.

**Conclusion:**

Patients diagnosed with CDI and no antibiotic exposures appear to be younger, frequently toxin EIA negative, and are more likely to have a community-onset CDI. Further research is required to determine if there are atypical risk factors within the community that place these patients at risk of developing a CDI.

**Disclosures:**

**Larry K. Kociolek, MD, MSCI**, Merck: Grant/Research Support **Curtis Donskey, MD**, Clorox: Grant/Research Support|Pfizer: Grant/Research Support **Stuart Johnson, M.D.**, Acurx Pharmaceuticals: Advisor/Consultant|Bio-K Plus International: Advisor/Consultant|Ferring Phamraceuticals: Advisor/Consultant **Dale N. Gerding, MD**, AstraZeneca: Advisor/Consultant|Destiny Pharma: Advisor/Consultant|Destiny Pharma: Licensed IP to Destiny|Sebela: Advisor/Consultant|Sebela: Licensed IP **Andrew M. Skinner, MD**, BioK plus: Advisor/Consultant|Ferring Pharmaceuticals: Advisor/Consultant|Recursion pharmaceutical: Advisor/Consultant

